# Profile of infective endocarditis observed from 2003 - 2010 in a single center in Italy

**DOI:** 10.1186/1471-2334-13-545

**Published:** 2013-11-15

**Authors:** Laurenzia Ferraris, Laura Milazzo, Davide Ricaboni, Cristina Mazzali, Giovanna Orlando, Giuliano Rizzardini, Marco Cicardi, Ferdinando Raimondi, Loredana Tocalli, Alessandro Cialfi, Paolo Vanelli, Massimo Galli, Carlo Antona, Spinello Antinori

**Affiliations:** 1 Department of Biomedical and Clinical Sciences “Luigi Sacco”, Università di Milano, “Luigi Sacco” Hospital, via G. B. Grassi, 74, 20157 Milano, Italy; 2 Third Division of Infectious Diseases, “Luigi Sacco” Hospital, Milano, Italy; 3 Second Division of Infectious Diseases, “Luigi Sacco” Hospital, Milano, Italy; 4 First Division of Infectious Diseases, “Luigi Sacco” Hospital, Milano, Italy; 5 Division of Internal Medicine, “Luigi Sacco” Hospital, Milano, Italy; 6 Intensive Care Unit, “Luigi Sacco” Hospital, Milano, Italy; 7 Microbiology Laboratory, “Luigi Sacco”, Milano, Italy; 8 Department of Cardiology, “Luigi Sacco” Hospital, Milano, Italy; 9 Cardiosurgery Division, “Luigi Sacco” Hospital, Milano, Italy

**Keywords:** Infective endocarditis, Echocardiography, Valvular disease, Prognostic factors, *Staphylococcus aureus*, Surgery

## Abstract

**Background:**

This study aimed to provide a contemporary picture of the epidemiologic, clinical, microbiologic characteristics and in-hospital outcome of infective endocarditis (IE) observed in a single center in Italy.

**Methods:**

We performed a retrospective study of patients with definite or probable IE observed at the “L. Sacco” Hospital in Milan, Italy, from January 1, 2003 through December 31, 2010.

**Results:**

189 episodes of IE in 166 patients were included. The mean number of incident IE in the study period was of 1.27 (range 0.59-1.76) cases per 1000 patients admitted. The median age of the cohort was 57 (interquartile range, 43-72) years, 63% were male and 62.5% had native valve IE. Twenty-six percent were active intravenous drug users (IVDU), 29% had a health care-associated IE and 5% chronic rheumatic disease. Twenty-nine percent of the cases occurred in patients affected by chronic liver disease and 19% in HIV positive subjects. *Staphylococcus aureus* was the most common pathogen (30%), followed by streptococci. The mitral (34%) and aortic (31%) valves were involved most frequently. The following complications were common: stroke (19%), non-stroke embolizations (25%), heart failure (26%) and intracardiac abscess (9%). Surgical treatment was frequently employed (52%) but in hospital mortality remained high (17%). Health care-associated IE and complications were independently associated with an increased risk of in-hospital death, while surgery was associated with decreased mortality.

**Conclusion:**

*S. aureus* emerged as the leading causative organism of IE in a University hospital in northern Italy. Our study confirmed the high in-hospital mortality of IE, particularly if health care associated, and the protective role of surgery.

## Background

The incidence of infective endocarditis (IE) has not decreased over the past 30 years, affecting between 2 and 6 per 100,000 subjects per year; however, in recent years, the epidemiology and clinical characteristics of IE have changed meaningfully [1-6]. Despite advances in medical and surgical therapy the associated mortality has remained between 10 and 30%, depending on the causative microorganism, the underlying conditions, and whether the infection occurred on native or prosthetic heart valve [7,8]. The emerging population at risk for IE consists of patients with health care-associated infections (acquired during hospitalization or following invasive procedures, mainly haemodialysis and chronic intravenous [IV] catheters), elderly people with degenerative valvulopathy, patients with prosthetic valves or intracardiac devices [2,6,9,10]. Other coexisting risk factors are diabetes mellitus and intravenous drug use [2,4,6,9-11]. The expansion of the elderly population with degenerative valve disease as well as the rise in staphylococcal infections suggest that IE will remain a significant health problem in the near future [12]. Oral streptococci are the predominant causative pathogen of IE in the general population [3,4,13], whereas *Staphylococcus aureus* and coagulase negative staphylococci (mainly *S. epidermidis*) are more often found in IVDU, in patients with prosthetic-valve endocarditis (PVE) or health care-associated IE [2,14-16]; furthermore group D streptococci (mainly *S. bovis*) are increasingly prevalent in elderly patients and are often associated with colon cancer [2-4,17,18]. The aim of this study was to describe a single-center cohort of patients with IE admitted in a tertiary care hospital in Milano, Italy, with particular emphasis on the clinical presentation, microbial aetiology, treatment and in-hospital mortality.

## Methods

### Design, patients and settings

A retrospective review of consecutive cases of IE was conducted at Luigi Sacco Hospital, a 550-bed university hospital, that is a referral center for infectious diseases (three wards) and cardiac surgery in the metropolitan area of Milano, Italy. The study was approved by the hospital Institutional Review Board (Comitato Etico per la sperimentazione clinica, Azienda Ospedaliera-Polo Universitario Luigi Sacco) and was conducted in accordance with the Declaration of Helsinki. All records from the years 2003–2010 were searched in the in-hospital database using discharge diagnosis according to the International Classification of Diseases, Ninth Revision (ICD-9) codes. Eighteen out of 207 records retrieved were excluded from the analysis: 4 patients did not have either definite or possible IE by Duke criteria, 14 patients had incomplete data or their clinical charts were unavailable. Of the remaining cases, 189 episodes occurring in 166 patients were evaluated.

IE was defined as definite or possible according to the modified Duke criteria [19]. Demographic data, clinical manifestations, treatment and in-hospital outcome information, laboratory and microbiology findings, were recorded into an electronic database. All clinical samples were collected as part of standard patient care.

### Echocardiographic data and case classification

Echocardiographic findings, inclusive of exam modality (transthoracic or transoesophageal), presence, size, location and mobility of vegetations; presence of valvular insufficiency; inferior vena cava size and caliber were recorded. Vital signs and pulmonary artery pressure were reported if available. Cases were also classified based on the valve involvement (tricuspid, mitral, aortic or pulmonary), whether native (NVE) or prosthetic (PVE) and according to the side (right, left or bilateral) of endocarditis. PVE were further defined as early and late recurrences, based on the timing of infection: within one year of the valve replacement or afterwards, respectively [20]. Infection of mitralic valvuloplasty and cardiac implantable electronic device-related IE (CIED-IE) that included pacemaker (PM) and implantable cardioverter defibrillator (ICD) infections, were integrated in the PVE group.

Health care-associated IE has been defined elsewhere [9-11]. Episodes of IE recurring more than 6 months after the end of therapy or within the 6 months period after therapy but caused by a different microorganism were considered reinfections; when IE was caused by the same microorganism within the 6 months period after the initial episode it was classified as a relapse.

### Statystical analysis

Categorical variables were expressed as number of cases (percentage); continuous variables were expressed as median ± interquartile range. Continuous data were analyzed using Wilcoxon non-parametric test, whereas for categorical variables Chi-square or Fisher exact tests were used for the analysis. Tests were two-sided and a *p* value less than 0.05 was considered statistically significant. Distribution of sex and age was evaluated on incident cases.

Incidence of endocarditis was calculated considering all hospitalized patients at L. Sacco Hospital from 2003 to 2010, and Chocran-Armitage trend test was performed.

Multiple linear regression analysis was performed to identify independent predictors of in-hospital mortality. A generalized linear model for binary response was used, and the presence of repeated measures for the same subject was handled through generalized estimating equations (GEE) method. An autoregressive model of covariances was considered. Age, sex and those variables, which presented a *p*-value less than 0.20 in univariate analyses, were entered in the final model. Analyses were performed using SAS® 9.2 (SAS Institute, Cary, NC, USA); GENMOD procedure was used for univariate and multivariate analyses.

## Results

A total of 189 episodes of IE were identified during the study period in 166 patients (104 males, 62 females). Baseline characteristics are shown in Table 1. The majority of cases (67%) were initially admitted to the Infectious Diseases Department; 27 (14%) to the Cardiac Surgery and cardiological-intensive care unit (ICU), 16 (9%) to the Internal Medicine wards, 7 (4%) to the general ICU, 6 (3%) to Cardiology ward and the remainder 6 (3%) to other wards.

**Table 1:** General characteristics of 166 patients with infective endocarditis (IE)

		**No. (%) of patients**			***p***
**Characteristic**		**TOTAL**	**Native valve**	**Prosthetic valve**	
		**(189 events, 166 patients)**	**(118 events, 111 patients)**	**(71 events, 55 patients)**	
**Age (years), median [IQR]**		57 [43–72]	53 [42–71]	66 [50–75]	0.01
**Male gender**		104 (63%)	72 (65%)	32 (58%)	0.4
**Time from onset of symptoms to admission**					
	< 7 days	69 (37%)	41 (35%)	28 (39%)	
	>7 and ≤21 days	42 (22%)	25 (21%)	17 (24%)	
	> 21 days	55 (29%)	34 (29%)	21 (30%)	1
	NA	23 (12%)	18 (15%)	5 (7%)	
**Co-morbidities**					
	Cancer	14 (7%)	13 (11%)	1 (1%)	0.01
	Hypertension	39 (21%)	21 (18%)	18 (25%)	0.2
	Acute/chronic renal failure	17 (9%)	6 (5%)	11 (15%)	0.02
	Ischemic cardiopathy	21 (11%)	9 (8%)	12 (17%)	0.05
	Diabetes mellitus	29 (15%)	17 (14%)	12 (17%)	0.6
	COPD	9 (5%)	4 (3%)	5 (7%)	0.2
	CLD	55 (29%)	41 (35%)	14 (20%)	0.03
	HIV infection	35 (19%)	27 (23%)	8 (11%)	0.05
	CD4 cells/μL [IQR]	268 [145–353]	248 [124–313]	447 [348–1814]	0.008
	HIV-RNA cp/mL (n 22)				
	<10000	14 (40%)	8 (30%)	6 (75%)	
	10000-100000	5 (14%)	4 (15%)	1 (13%)	0.11
	>100000	4 (11%)	4 (15%)	0	
	NA	12 (34%)	11 (41%)	1 (13%)	
	HAART at IE				
	No	21 (60%)	15 (56%)	6 (75%)	0.2
	Yes	14 (40%)	12 (44%)	2 (25%)	
Data are median [interquartile range] or numbers (%).					

*NA*, Not available; *COPD*, Chronic obstructive pulmonary disease; *CLD*, Chronic liver disease; *HAART*, Highly active antiretroviral therapy.

**Figure 1:**
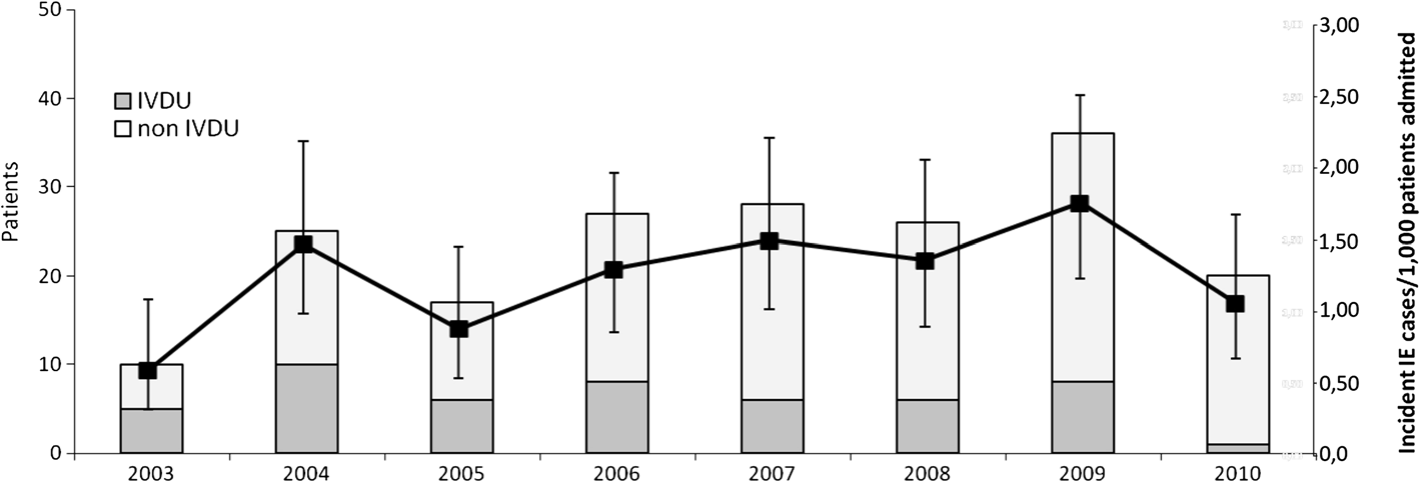
Incident cases of IE observed at the L. Sacco Hospital in the period 2003–2010: p = 0.067 according to Cochran-Armitage trend test.

After excluding prevalent cases, the mean number of incident IE over the period 2003–2010 at Luigi Sacco Hospital was 1.27 (range: 0.59-1.76) cases per 1000 admissions, without a statistically significant trend (*p* = 0.0671) (Figure 1). One hundred and forty-four cases (76%) were diagnosed as definite IE and the remainder as probable IE.

### Clinical and laboratory characteristics

Underlying disorders and predisposing conditions are summarized in Tables 1 and 2. Seventy-one percent of IE were classified as community acquired, 29% as health care-associated. Left-sided IE occurred in 140 cases (74%), right-sided IE in 44 (23%) cases; 5 episodes (3%) involved both heart sides. Seventy-one events interested prosthetic valves (72% biologic valves). Aortic valve was affected in 65 cases (34%), mitral valve in 59 cases (31%), tricuspid valve in 38 cases (20%). Multiple valve involvement was more commonly observed in native rather than prosthetic valves (21/118 cases [18%] *vs* 3/71 cases [4%], respectively; *p* = 0.007).

**Table 2:** Underlying disorders and predisposing conditions in patients with infective endocarditis (IE)

	**No. (%) of patients**			
	**TOTAL**	**Native valve**	**Prosthetic valve**	***p***
Current IV drug use	50 (26%)	35 (30%)	15 (21%)	0.2
Steroid therapy	5 (3%)	3 (3%)	2 (3%)	1
Previous IE	36 (19%)	12 (10%)	24 (34%)	<0.0001
Health care associated IE*	54 (29%)	33 (28%)	21 (30%)	0.3
Congenital heart disease	6 (3%)	6 (5%)	0	0.09
Rheumatic heart disease	9 (5%)	8 (7%)	1 (1%)	0.2
Pacemaker	11 (6%)	2 (2%)	9 (13%)	0.003
Data are median [interquartile range] or numbers (%).				

*Health care associated IE: chronic IV access/hemodialysis: 7/189 (4%); invasive procedures before IE (dental, urological and gastrointestinal procedures): 7/189 (4%) hospitalized in an acute care hospital before IE 38/189 (20%); resident in a nursing home or in a long-term care facility 2/189 (1%).

Fever was the main sign of clinical presentation observed in 81% of cases and a new onset or a worsening cardiac murmur was reported in 79% of cases. Notably, 29% of patients diagnosed to have IE in our cohort had been admitted after a long history of fever (>21 days).

The mean WBC count on admission was 9670 cells/μL (IQR: 7190–12910 cells/μL), haemoglobin was 10.5 g/dL (IQR: 9.2-12.1 g/dL), creatinine 0.9 mg/dL (IQR: 0.7-1.48 mg/dL), lactic dehydrogenase (LDH) 345 U/L (IQR: 244.5-474.5 U/L) and C reactive protein (PCR) 54.7 mg/dL (IQR: 9.75-146.75 mg/dL). No difference in laboratory findings were found between NVE and PVE, except for creatinine (0.84 [IQR: 0.7-1.2] mg/dL and 1.07 [IQR: 0.8-1.83] mg/dL, respectively, p = 0.02), LDH (340 [IQR: 219–449] U/L and 358 [IQR: 259–537] U/L, respectively, p = 0.04) and PCR (23.9 [IQR: 7.6-106] mg/dL *vs* 67 [IQR: 33.1-179] mg/dL, respectively, p = 0.003).

### Relapses and reinfections

Throughout the study period 15 patients presented 23 recurrences (17 reinfections and 6 relapses). Twelve recurrences out of 23 occurred in 7 active intravenous drug users (IVDU), 5 of whom were HIV positive, and 3 were classified as relapses, whereas 9 as reinfections. In 6/15 patients recurrence occurred in native valves, while in 9/15 a prosthetic valve was involved. All the recurrences affected the same valve of the first IE episode: tricuspid (45%) and aortic (33%) disease prevailed, followed by mitral involvement (22%). A different microorganism was isolated at any reinfection in 87% of cases: staphylococci were the most common causative pathogens (44%), followed by enterococci (26%), by streptococci (22%) and by Gram negative bacteria (*Pseudomans* spp: 8%). The median time from the first episode of IE to the recurrence was 1 year.

### Echocardiographic findings

Echocardiographic description was available in 92% of episodes, as 8% of subjects were referred to our center with a diagnosis performed in other institutes. Sixty-seven percent of patients underwent both transthoracic (TTE) and transoesophageal (TEE) echocardiography. Ninety-one percent of cases (158) had echocardiographic evidence of vegetation, with significant valvular regurgitation observed in 65% (113). The destruction of valvular tissue was evidenced in 25% of cases (44), 70% of which (31/44) were observed in NVE (fistula/perforation/rupture), with a prevalence of 28%, while 30% (13/44) occurred in PVE, with a prevalence of dehiscence/paravalvular leakage in the latter group of 20%. Moderate to severe regurgitation was more frequently detected in NVE (84/111 cases [76%] *vs* 28/65 cases [43%]; p = 0.0001).

### Microbiology

**Figure 2:**
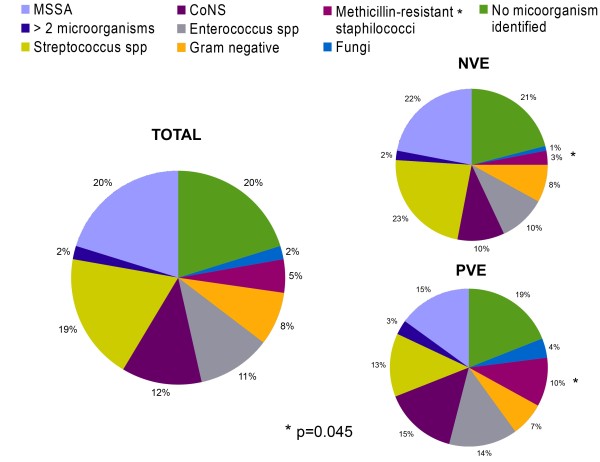
**Microorganisms cultured according to the different type of IE.***MSSA*: Methicillin-sensitive *Staphylococcus aureus; CoNS*: Coagulase-negative Staphylococci*;* Methicillin-resistant staphilococci: MRSA (methicillin-resistant *Staphylococcus aureus),* methicillin-resistant *Staphylococcus epidermidis. Streptococcus* spp. includes: Viridans group Streptococci (overall 41.7%); *S. bovis* (overall 25%); other Streptococci (overall 33.3%).

Blood cultures were performed in 186 of the 189 episodes (98%). Of the 37 patients (20%) with negative blood cultures, 24 (65%) had received antibiotics before blood collection. *Staphylococcus aureus* was the most frequently isolated pathogen in our series (44/149 episodes of IE, 30%); 16% of those strains were methicillin-resistant *Staphylococcus aureus* (MRSA). *Streptococcus* spp.  was the second most prevalent organism (19%) (viridans group streptococci 41.7%, *S. bovis* 25%, other streptococci 33.3%), followed by coagulase-negative staphylococci (CoNS 12%). No noticeable change was observed in either distribution or antibiotic sensitivity of organisms during the study period (data not shown). Microbial aetiology by type of IE is reported in Figure 2.

No association between staphylococcal aetiology and health care-associated IE was found (*p* = 0.6; data not shown), whereas IE due to methicillin-resistant staphilococci were significantly more likely to occur in PVE patients than in NVE (*p* = 0.045). In addition, S*taphylococcus aureus* was significantly more frequently isolated in right-sided than in left-sided IE (18/44 cases, 41% *vs* 17/140 cases, 18% respectively; *p* < 0.0001) and in IVDU compared to non-IVDU (19/50 cases, 38% *vs* 18/136 cases, 13% respectively; *p* = 0.0002), while streptococcal strains were more responsible for left-sided than right-sided IE (32/140 cases, 23% *vs* 3/44 cases, 7% respectively; *p* = 0.02) and for non-IVDU- compared to IVDU-associated cases (31/136 cases, 23% *vs* 5/50 cases, 10% respectively; *p* = 0.05). We found that *Enterococcus* spp. were significantly associated with left-sided IE (*p* = 0.05) and CoNS infections were more likely to occur in early recurrence of IE rather than in late recurrence (7/24 cases, 29% *vs* 2/37 cases, 5% respectively; *p* = 0.02).

### Characteristics of IE in the HIV-positive population

Thirty-five episodes of IE occurred in 27 HIV-infected patients: most of them were male (81%) with a median age of 42 (IQR 37–47) years and the major route of HIV transmission was intravenous drug use (23; 85%). Virological and immunological characteristics as well as antiretroviral regimens at the time of IE are summarized in Table 1. Compared with the HIV-negative group, the HIV-infected patients had a higher incidence of right-sided IE (48% *vs* 17%, *p* = 0.0001) and MSSA-sustained infections (34% *vs* 17%, *p* = 0.02), mirroring the higher prevalence of IVDU (85% *vs* 10% in HIV-negative, *p* = 0.0001).

### Complications, treatment and outcomes

Complications, treatment choice and in-hospital outcome by type of IE are shown in Table 3. All patients were treated with antibiotics for 4 to 6 weeks. The association of three or more antibiotics was observed more frequently in PVE than NVE (*p* = 0.02). Overall, 99 patients (52%) underwent surgery, which was performed on an elective basis in most patients (84%). The median time between hospital admission and surgery was 22 days (IQR 12–38).

**Table 3:** Complications, treatment and outcome of IE episodes

		**No. (%)**			
**Characteristic**		**TOTAL**	**Native valve**	**Prosthetic valve**	**p**
		**(189 cases)**	**(118 cases)**	**(71 cases)**	
Complications					
	Embolizations*	83 (44%)	56 (47%)	27 (38%)	0.4
	Heart failure	49 (26%)	32 (27%)	17 (24%)	0.6
	Arrhythmias	40 (21%)	28 (24%)	12 (17%)	0.3
	Intracardiac abscess	15 (9%)	6 (5%)	9 (15%)	0.04
	Renal failure	12 (6%)	6 (5%)	6 (8%)	0.4
	Septic shock	8 (4%)	2 (2%)	6 (8%)	0.05
Medical treatment					
	Monotherapy	18 (10%)	13 (11%)	5 (7%)	0.02
	Dual therapy	115 (62%)	78 (68%)	37 (53%)	
	≥three drug therapy	52 (28%)	24 (21%)	28 (40%)	
Surgical treatment					
	Medical and surgical	99 (52%)	65 (55%)	34 (47%)	0.3
	Multiple valve procedures	24 (24%)	18 (15%)	6 (8%)	0.2
Surgical treatment timing					
	Emergency (≤48 h)	4 (4%)	3 (5%)	1 (3%)	0.4
	Urgent (>48 h and ≤7 days)	12 (12%)	9 (14%)	3 (9%)	
	Elective (> 7 days)	83 (84%)	53 (81%)	30 (88%)	
In-hospital mortality		32 (17%)	16 (14%)	16 (23%)	0.1
	Pre-operative	20 (63%)	11 (69%)	9 (56%)	0.8
	Intra-operative	2 (6%)	1 (6%)	1 (6%)	
	Post-operative	10 (31%)	4 (25%)	6 (38%)	

*Embolizations: Stroke (35, 19%); Non-Stroke (48, 25%) [Pulmonary (21), Cutaneous (5); Upper/lower extremities (3); Myocardial infarction (1); Splenic (12); Bone (6)].

In-hospital mortality was 17% (32 of 189 cases). Causes of in-hospital death were septic shock with multiorgan failure (10/32, 31%), heart failure (10/32, 31%), stroke (4/32, 13%), surgery-related complications (8/32, 25%), such as hemorrhagic shock, arrhythmias, metabolic acidosis and systemic embolism. We did not find any statistically significant difference between early (within 7 days) and elective surgery regarding in-hospital mortality (*p* = 0.3; data not shown).

Table 4 shows the results of the regression modeling for in-hospital mortality. Among all the variables associated with increased mortality at univariate model, only health care-associated IE (OR = 3.35, *p* = 0.01) and the onset of any complication (OR = 5.58, *p* = 0.003) were found to be independently associated with an increased risk of in-hospital death in the multivariate analysis, while surgery was independently associated with higher in-hospital survival (OR 0.38, *p* = 0.04).

**Table 4:** Results of multivariable regression modeling of associations with in-hospital death

**VARIABLE**	**AOR (95% CI)**	**p value**
Age in 10 y intervals	1.26 (0.95-1.69)	0.1
Female sex	0.60 (0.20-1.78)	0.4
PVE early recurrence	0.88 (0.30-2.59)	0.8
PVE late recurrence	2.12 (0.80-5.58)	0.1
Intravenous drug use	0.60 (0.14-2.57)	0.5
Health care associated IE	3.35 (1.27-8.78)	0.01
CoNS – associated IE	0.50 (0.09-2.78)	0.4
*Staphylococcus aureus*	3.83 (0.95-15.44)	0.058
Viridans group streptococci	1.26 (0.33-4.77)	0.7
Others*	1.98 (0.58-6.80)	0.3
Mitral valve involvement	1.33 (0.53-3.30)	0.5
Complications**	5.58 (1.79-17.31)	0.003
Surgery	0.38 (0.16-0.94)	0.04

*CoNS*, Coagulase-negative staphilococci; *MSSA*, Methicillin-sensitive *Staphylococcus aureus*; *MRSA*, Methicillin-resistant *Staphylococcus aureus*; *PVE*, prosthetic valve endocarditis.

*Others: *Enterococcus* spp, Fungi/Yeast, Gram-negative bacteria, polymicrobial.

**Complications: heart failure, renal failure, stroke, non stroke embolization, intracardiac abscess, septic shock, arrhythmias.

## Discussion

Our study has several limitations and potential biases, being retrospective and performed in a single referral center with the largest Department of Infectious Diseases in the city of Milano. Therefore, as for most of institutional surveys, it cannot provide a representative picture of IE incidence in the general population. Indeed, about 67% of all cases were hospitalized into the Infectious Diseases Department, a figure that is lower in comparison with the 90% observed in a Japanese study [21]. It is notable that in our series about 30% of all patients had a long duration of symptoms (more than 3 weeks) before hospital admission and, more notable, without any difference between those with NVE in comparison with PVE. Since the majority of cases were admitted to investigate a long-lasting fever, we can speculate that there is a low awareness of the disease among general practitioners even in the high risk population.

The incidence of IE in our cohort was of 1.27/1000 admissions without a significant increase from 2003 to 2010. The median age (57 years), gender (63% males) and prevalence of definite IE were in agreement with data reported from recent reports [3,6,21]. Moreover, consistently with recent studies conducted in Western countries [9,18,22], about 30% of all episodes of IE in our cohort were associated with the health care system, probably reflecting the improved longevity with a concomitant increase of comorbidities, degenerative valvular alterations and health care-associated infections. It is worth noting that HIV infection was recorded in 19% of episodes of IE in our series. This is a quite higher prevalence in comparison with a recent multicenter Italian study [23] and a single center study from Spain [24]. Since in our hospital more than 6000 HIV-positive patients are regularly followed as outpatients, the high prevalence of concomitant HIV infection is not unexpected among patients with a diagnosis of IE [25]. HIV-positive patients were younger (*p* = 0.0001), more frequently male (*p* = 0.03), had higher frequency of right-sided IE (*p* = 0.0001) and *S. aureus* was more commonly identified in comparison with HIV-negative subjects. All these aspects reflect the main risk factor of acquisition of both HIV and IE in our population, that is intravenous drug use.

Excluding HIV infection, chronic liver disease (CLD), diabetes, and hypertension were the most commonly observed comorbidities in our cohort. About one third of our patients had CLD with a statistically significant difference among patients with NVE in comparison with those affected by PVE (*p* = 0.03). Both CLD and diabetes have been previously reported as important comorbidities and as independent predictors of mortality [26,27]. At variance with the above cited studies, in our analysis neither CLD nor diabetes were found to be associated with in-hospital mortality.

Our study confirms recent data on the leading role of *S. aureus* as the causative organism of IE either in Italy and France [22,23]. The reported worldwide increase in the incidence of IE due to *S. aureus* has been associated with the widespread use of medical devices and procedures that is also responsible of health care-associated IE. However, in our experience it can be at least partly attributed to an overrepresentation of IVDU, who carry a high risk of percutaneous *S. aureus* exposure. Similarly, the high incidence of recurrences observed in our cohort (12% of cases), with 52% occurring in IVDU is consistent with the high prevalence of IVDU among our patients. Moreover, it cannot be ruled out that the three cases defined as relapses in IVDU might have actually been a reinfection resulting from an ongoing exposure to drugs injections.

Two disappointing findings of our study were the fact that 2% of patients did not have blood cultures performed and the high incidence of negative blood cultures (20%). The first issue is essentially a cultural problem that needs to be addressed by educational programs. The high proportion of blood culture negative IE might be explained in part by the fact that 64% of these patients had received antibiotics by the time of blood sample taking. The proportion of negative blood cultures ranged from 5 to 10% in recent surveys from multicenter cohorts [3,6,23] that involved selected tertiary care institutions. However, in the Euro Heart Survey, a prospective study involving 92 centers from 25 European countries and in an Italian population-based surveillance, the incidence of negative blood cultures was 14% and 19.3%, respectively [26,28]. We believe that the microbiology diagnosis can be improved by implementing either serological testing or molecular methods in order to detect rare and fastidious bacteria and fungi that are difficult to grow [24,29-31]. In agreement with other studies, heart failure and non-stroke embolism were the most common clinical complications observed in our experience [21,23,24]. As expected, intracardiac abscess and septic shock were more frequently observed among patients with PVE [32,33]. In our cohort complications and health care-associated IE were the only independent variables associated with in-hospital mortality in a multivariable model.

Finally, in-hospital mortality in our cohort was 17% that is similar to the death-rate reported in France, in the Italian Study on Endocarditis and in a European survey and ranging from 12.6% to 16.6% [3,23,34]. In agreement with a recent prospective analysis of the Italian Study on Endocarditis, we found that surgery had a protective effect with respect to in-hospital mortality [23].

## Conclusions

In conclusion, despite the availability of new and potent antibiotics, modern echocardiography, and advanced surgical techniques, IE is still associated with a high mortality. This study provides a comprehensive, single-center picture of IE, and, therefore, despite its limitations, may allow to identify critical issues related to this disease and possibly contributes to the improvement of the clinical management of IE in our hospital.

## Competing interests

The authors declare that they have no competing interests.

## Authors’ contributions

LF, participated in patients selection, created the database and data analysis and drafted the manuscript. LM participated in data analysis and drafted the manuscript. DR contributed to patients selection and created the database. CM carried out the statistical analysis. GO, MC, FR contributed to patients selection and made critical review of the paper. LT created the microbiological database. AC performed the echocardiographic studies and participated in the database analysis. PV, CA performed cardiac surgery and participated in the database analysis. GR, MG participated in the study design and made critical review of the paper. SA conceived the study design, made data analysis and drafted the manuscript. All authors read and approved the final manuscript.

## Pre-publication history

The pre-publication history for this paper can be accessed here:

http://www.biomedcentral.com/1471-2334/13/545/prepub
